# Association of CDKAL1 RS10946398 Gene Polymorphism with Susceptibility to Diabetes Mellitus Type 2: A Meta-Analysis

**DOI:** 10.1155/2021/1254968

**Published:** 2021-12-24

**Authors:** Ning Xu, Ting-Ting Zhang, Wen-Jia Han, Li-Ping Yin, Nan-Zheng Ma, Xiu-Yan Shi, Jiang-Jie Sun

**Affiliations:** ^1^School of Pharmacy, Anhui Medical University, Hefei, Anhui 230032, China; ^2^School of Dentistry, Anhui Medical University, Hefei, Anhui 230032, China; ^3^Medical Examination Center, The First Affiliated Hospital of Anhui Medical University, Hefei 230022, China; ^4^Anhui Medical University, Affiliated Hospital, Nationalities, Hefei, Anhui 230032, China; ^5^Nanjing Prevention and Treatment Center for Occupational Diseases, Nanjing 23100, China; ^6^Health Management College, Anhui Medical University, Hefei, Anhui 230032, China

## Abstract

**Background:**

Diabetes is one of the common chronic diseases in which susceptibility is determined by a combination of genetic and environmental factors, and more than 90% of diabetic patients are diabetes mellitus type 2 (T2DM). The existing studies on the association between CDKAL1 rs10946398 gene polymorphism and susceptibility to type 2 diabetes are inconsistent across populations.

**Aim:**

We aim to explore the association between CDKAL1 rs10946398 gene polymorphism and susceptibility to type 2 diabetes in different populations.

**Methods:**

We examined all studies before June 12, 2021, that associated CDKAL1 rs10946398 with T2DM. Heterogeneity was assessed by meta-analysis of allelic inheritance models (A vs. C), dominant inheritance models (AA vs. AC+CC), and recessive inheritance model (AA+AC vs. CC); *I*^2^ was used to assess the heterogeneity (if *I*^2^ < 50%, the fixed-effects model was used; if *I*^2^ ≥ 50%, the random-effects model was used for data consolidation); correlation was judged by a forest map; potential publication bias was tested by the Egger test (*p* > 0.05 indicates that there is no publication bias).

**Results:**

Fourteen data totaling 30288 subjects, including 19272 controls and 11016 patients with T2DM, met our inclusion criteria. In the Asian population, the differences were statistically significant (*p* < 0.01) for dominant genetic model (OR = 0.75, 95%CI = 0.64-0.88, *p* = 0.0003). But the allelic effect model (OR = 0.87, 95%CI = 0.75-1.02, *p* = 0.08) and the recessive genetic model (OR = 0.85, 95%CI = 0.66-1.10, *p* = 0.23) were not statistically significant (*p* > 0.01). In the non-Asian population, the differences were statistically significant (*p* < 0.01) for the allelic effect model (OR = 0.83, 95%CI = 0.77-0.88, *p* < 0.00001), the dominant model (OR = 0.79, 95%CI = 0.72-0.87, *p* < 0.00001), and the recessive model (OR = 0.78, 95%CI = 0.70-0.87, *p* < 0.0001).

**Conclusion:**

In this study, CDKAL1 RS10946398 was positively associated with T2DM, but the association was different in Asian populations.

## 1. Introduction

According to the World Health Organization (WHO), approximately 3.4 million people died from developing diabetes in 2004, and it predicts that the number of diabetes deaths will double between 2005 and 2030. The International Diabetes Federation predicts that the global prevalence of diabetes will reach 642 million cases by 2040 (International Diabetes Federation, 2015), with type 2 diabetes accounting for more than 90% of diabetics [1].

Type 2 diabetes mellitus (T2DM), formerly known as non-insulin-dependent or adult-onset diabetes mellitus, is a type of diabetes mellitus. It is caused by poor insulin action which is the relative lack of insulin in patients, and its susceptibility is determined by both genetic and environmental factors [2]. In the context of increasing morbidity and mortality of T2DM, it is of great significance to study the pathogenesis of T2DM.

Previous studies have shown that China [3–6] and other Populations of Asian countries' CDKAL1 RS10946398 locus mutation was significantly associated with T2DM [1, 7–10]. The United States [11, 12], Russia [13, 14], Mexico [15], and other non-Asian populations of CDKAL1 RS10946398 were also significantly associated with T2DM. It is noteworthy that a variant of the CDKAL1 RS10946398 locus in the population of the Asian country of the United Arab Emirates may not be directly associated with the development of T2DM [1]. These show that CDKAL1 rs10946398 locus variants play different roles in different study populations. Therefore, it is of great significance to study the relationship between CDKAL1 rs10946398 locus variation and T2DM susceptibility in different populations.

### 1.1. Retrieval Strategy

An advanced search of the literature search library was conducted by using “T2DM CDKAL1” and “CDKAL1 rs10946398” as key to search terms in the China National Knowledge Infrastructure (CNKI), PubMed, and WanFang digital databases, with the last search conducted on June 12, 2021.

### 1.2. Inclusion and Exclusion Criteria

The following studies were included [16, 17]:

1. Case-control studies focus on the association between the CDKAL1 rs10946398 polymorphism and T2DM in adults
2. Patients were randomly selected with no special restrictions on gender, family history, etc.
3. Studies provide accurate control and case group data sources
4. The data provided in the study report were statistically significant. The study results had specific OR values, 95% CI
5. Studies met the diagnostic criteria of T2DM published by the World Health Organization (WHO) in 2019, and the control group all met the law of H-W genetic balance

The following studies were excluded:

1. There were only case groups or a lack of sufficient controls
2. Statistical data are erroneous or there are significant differences in the statistics of the same study in different literatures
3. The overall sample size is insufficient
4. Literature reviews and case reports were excluded

### 1.3. Data Extraction

**Figure 1:**
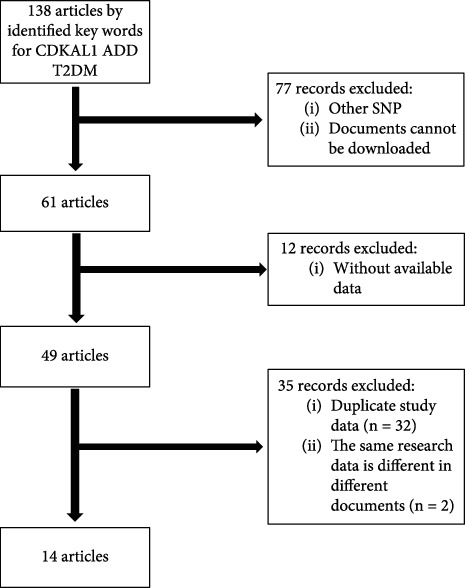
Literature screening flow chart.

Two investigators independently read the literature and extracted information from the eligible literature based on exclusion and inclusion criteria. In case of ambiguity, a consensus was reached on whether to extract the paper data through discussion with the third investigator. For each paper, the following information was collected: (i) author's name, (ii) year of publication, (iii) ethnicity and country of the study population, (vi) number of included cases and controls, and (vii) genotype data [3]. The literature screening process is shown in Figure 1.

### 1.4. Statistical Methods

Review Manager 5 software was used to complete the meta-analysis. Stata software was used to complete the Egger test.

## 2. Results

### 2.1. Baseline Characteristics of Included Studies

We obtained articles on the relationship between CDKAL1 rs10946398 diversity and T2DM susceptibility from PubMed and CNQ. After reading the title, year, author, and abstract of the papers, we conducted the first screening. The second screening was performed by reading the full text and analyzing whether the data was statistically significant. Finally, 14 literatures were included. A total of 14 datasets were obtained for meta-analysis by reading through the full text to filter the data required for recording. A total of 30288 subjects were included in the meta-analysis, including a total of 11016 in the T2DM patient group and 19272 in the control group. Eight of the datasets were from the Asian study population: 3 from China, 1 from India, 1 from Korea, 1 from Japan, 1 from Iran, and 1 from the United Arab Emirates; 6 were from the non-Asian study population: 3 from the USA, 2 from Russia, and 1 from Mexico. Information on the first author, study year, sample size, ethnicity, BMI, mean age of control and case groups, and risk allele frequency for each study is shown in Table 1.

## 3. Results of Meta-Analysis

In the evaluation of the relationship between the CDKAL1 rs10946398 gene and T2DM susceptibility, a total of 14 studies were included in the meta-analysis after literature data search, screening, and verification. In order to analyze the association between CDKAL1 rs10946398 polymorphism and susceptibility to T2DM, we analyzed the relationships between A and C alleles, AA+AC and CC genotypes, AA and AC+CC genotypes in T2DM patients and controls. Since 8 studies were from Asia and 6 were from non-Asia, we stratified the Asian and non-Asian populations.

We examined heterogeneity separately for the study populations, using *I*^2^, to assess the magnitude of heterogeneity (if *I*^2^ < 50%, a fixed-effects model was used; if *I*^2^ ≥ 50%, a random-effects model was used to combine the data). Because our data were randomly selected and we wanted to reflect the overall situation with a small sample size, only the allelic genetic model and the recessive genetic model in non-Asian populations show that *I*^2^ < 50%, so we used the random-effects model (see Table 2).

**Figure 2:**
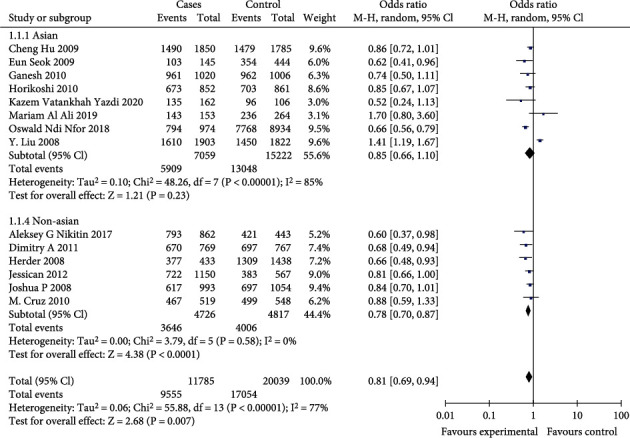
Forest plot of meta-analysis of the A vs. C allele model associated with T2DM at CDKAL1 rs10946398 locus.

**Figure 3:**
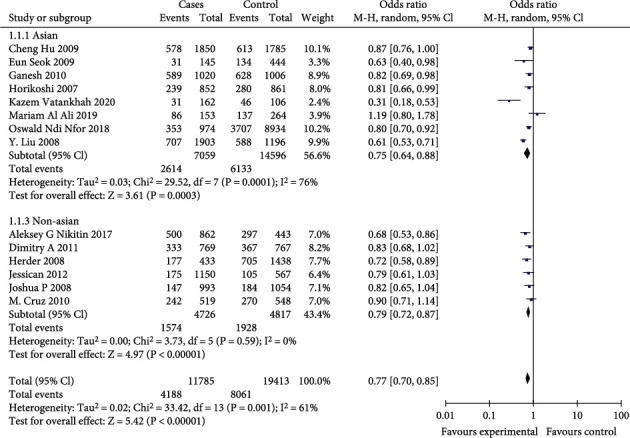
Meta-analysis of a T2DM-associated AA vs. AC+CC genotype model at CDKAL1 RS10946398 locus forest map.

**Figure 4:**
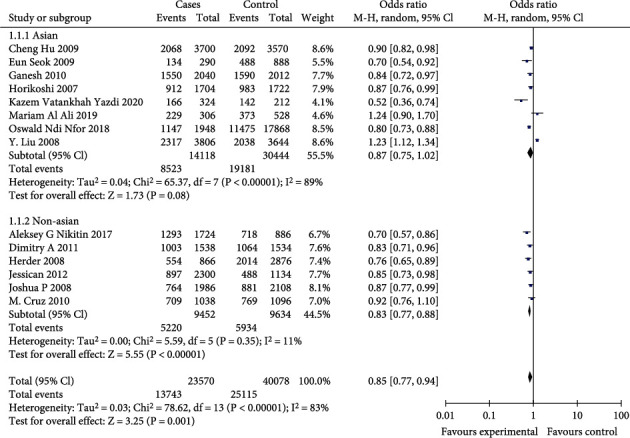
Meta-analysis of a T2DM-associated AA+AC vs. CC genotype model at CDKAL1 RS10946398 locus forest map.

In the total population, the differences were statistically significant (*p* < 0.01) for the allelic genetic models (OR = 0.85, 95%CI = 0.77-0.94, *p* = 0.001), the dominant genetic models (OR = 0.77, 95%CI = 0.70-0.85, *p* < 0.00001), and the recessive genetic models (OR = 0.81, 95%CI = 0.69-0.94, *p* = 0.007). The results are shown in Figures 2–4.

In the Asian population, the differences were statistically significant (*p* < 0.01) for dominant genetic model (OR = 0.75, 95%CI = 0.64-0.88, *p* = 0.0003). But the allelic effect model (OR = 0.87, 95%CI = 0.75-1.02, *p* = 0.08) and the recessive genetic model (OR = 0.85, 95%CI = 0.66-1.10, *p* = 0.23) were not statistically significant (*p* > 0.01). The results are shown in Figures 2–4.

In non-Asian populations, the differences were statistically significant (*p* < 0.01) for the allelic genetic model (OR = 0.83, 95%CI = 0.77-0.88, *p* < 0.00001), the dominant genetic model (OR = 0.79, 95%CI = 0.72-0.87, *p* < 0.00001), and the recessive genetic model (OR = 0.78, 95%CI = 0.70-0.87, *p* < 0.0001). The results are shown in Figures 2–4.

### 3.1. Publication Bias

**Figure 5:**
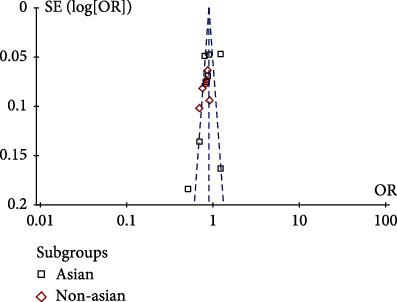
A vs. C allelic funnel plot.

**Figure 6:**
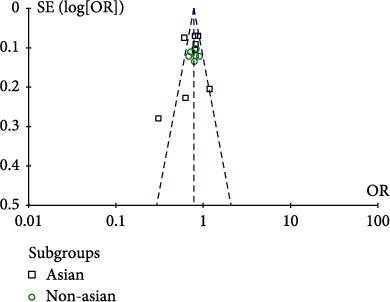
AA vs. AC+CC genotype funnel plot.

**Figure 7:**
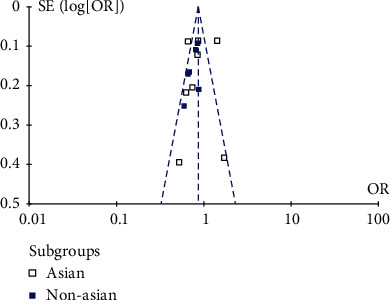
AA+AC vs. CC genotype funnel plot.

We used Stata software for the Egger test, and the *p* values of allelic inheritance models (A vs. C), recessive inheritance model (AA+AC vs. CC), and dominant inheritance models (AA vs. AC+CC) were 0.114, 0.307, and 0.304, respectively, which were greater than 0.05, indicating that there was no publication bias. What is more, according to the symmetry of the funnel plot, the existence of publication bias can also be judged. The results are shown in Figures 5–7; it can be found that all points in the funnel plot are distributed symmetrically along both sides of the midline, so there is no bias.

## 4. Discussion

According to a large number of genome-wide association analyses (GWAS), CDK5 regulation-related protein 1-LIAK 1 (CDKAL1) gene under the action of high glucose toxicity will increase the body's demand for insulin, and pancreatic *β* cells continue to be activated, which may inhibit the activity of CDK5 in pancreatic *β* cells. Insulin secretion is reduced by lowering the expression of insulin genes [18, 19]. Because mutations in CDKAL1 may lead to impaired insulin secretion, thus, it increases the risk of T2DM, and CDK5 regulates the related protein 1-LIAK 1 (CDKAL1) gene which is one of the most repeatable risk genes in T2DM [20]. In particular, SNPs rs10946398 and rs7754840 of CDKAL1 have the strongest correlation with T2DM [20].

To study the relationship between the variation of CDKAL1 RS10946398 locus and the susceptibility to T2DM in different populations, 14 sets of data were finally used for meta-analysis through data investigation and screening, and 13 sets of data showed that the CDKAL1 RS10946398 locus was significantly correlated with the incidence of T2DM; for example, a study by Nfor et al. showed a significant association between CDKAL1 RS10946398 and T2DM in Taiwanese. CC carriers were more associated with T2DM than AC carriers, and C allele carriers were more associated with type 2 diabetes than A allele carriers [6]. A study by Herder et al. found that CDKAL1rs10946398 was significantly associated with impaired glucose metabolism or *β* cell function. CDKAL1rs10946398 also plays an important role in the pathogenesis of T2DM in the detected Russian population [12]. Only one set of data showed that CDKAL1 RS10946398 locus was not significantly associated with the pathogenesis of T2DM. The study by Al Ali et al. showed that the CDKAL1 RS9939609 variant in the United Arab Emirates population may not be directly related to the development of T2DM [1]. Therefore, the role of CDKAL1 rs10946398 locus variation in different study populations is different.

In this study, a meta-analysis of the included 14 groups of data concerning the CDKAL1 rs10946398 locus and T2DM was performed by analyzing allelic models (A vs. C), recessive genetic models (AA+AC vs. CC), and dominant genetic models (AA vs. AC+CC) in T2DM patients and controls. Of the 30288 subjects, including 19272 controls and 11016 T2DM patients, we found that CDKAL1 RS10946398 gene polymorphism locus is associated with type 2 diabetes mellitus in different ethnic groups, and the degree of correlation is different in different genetic models.

In the Asian population, the differences were statistically significant (*p* < 0.01) for the dominant genetic model (OR = 0.75, 95%CI = 0.64-0.88, *p* = 0.0003). But the allelic effect model (OR = 0.87, 95%CI = 0.75-1.02, *p* = 0.08) and the recessive genetic model (OR = 0.85, 95%CI = 0.66-1.10, *p* = 0.23) were not statistically significant (*p* > 0.01). The risk ratio of the A allele was higher than that of the C allele. In the non-Asian population, the differences were statistically significant (*p* < 0.01) for the allelic effect model (OR = 0.83, 95%CI = 0.77-0.88, *p* < 0.00001), the dominant model (OR = 0.79, 95%CI = 0.72-0.87, *p* < 0.00001), and the recessive model (OR = 0.78, 95%CI = 0.70-0.87, *p* < 0.0001). The risk ratio of the A allele was higher than that of the C allele [21].

We used 14 sets of data for meta-analysis of the locus genetic model (A vs. C) recessive models (AA+AC vs. CC) and dominant models (AA vs. AC+CC). Except for the Asian allelic effect model and recessive gene model (*p* > 0.01), other models were statistically significant (*p* < 0.01). CDKAL1rs10946398 could significantly increase the risk of T2DM in the allele model of Asian and in all models of non-Asian. But this result cannot be attributed to differences in ethnicity; it could also be due to the small sample size. In conclusion, the CDKAL1 rs10946398 gene variant may increase the susceptibility to T2DM.

## Figures and Tables

**Table 1:** Association of CDKAL11 rs10946398 polymorphism with T2DM susceptibility.

First author	Region	Race	BMI		Age (yr)		Controls						Cases					
			Controls	Cases	Controls	Cases	N	AA	AC	CC	A	C	N	AA	AC	CC	A	C
Horikoshi (2007)	Asian	Japanese	23.8 ± 3.7	24.3 ± 3.9	69.5 ± 6.8	63.1 ± 9.5	861	280	423	158	983	739	852	239	434	179	912	792
Joshua P (2008)	Non-Asian	American	NA	NA	NA	NA	1054	184	513	357	881	1227	993	147	470	376	764	1222
Y. Liu (2008)	Asian	Chinese	24.5 ± 3.2	25.3 ± 3.4	58.1 ± 9	63.8 ± 9	1822	588	862	372	2038	1606	1903	707	903	293	2317	1489
Herder (2008)	Non-Asian	American	27.7 ± 4.3	30.9 ± 5.0	61.6 ± 9.7	59.9 ± 7.9	1438	705	604	129	2014	862	433	177	200	56	554	312
Eun Seok (2009)	Asian	South Korea	NA	NA	37.4 ± 9.3	42.6 ± 9.1	444	134	220	90	488	400	145	31	72	42	134	156
Cheng Hu (2009)	Asian	Chinese	23.57 ± 3.25	24.04 ± 3.51	57.39 ± 12.37	61.21 ± 12.62	1785	613	866	306	2092	1478	1850	578	912	360	2068	1632
M. Cruz (2010)	Non-Asian	Mexico	27.50 ± 3.55	29.25 ± 4.76	43.60 ± 6.63	53..44 ± 7.42	548	270	229	49	769	327	519	242	225	52	709	329
Ganesh (2010)	Asian	India	Women 24.90 (21.10–28.60) Men 23.20 (20.20–25.70)	Women 26.70 (24.20–29.20) Men 23.80 (22.00–26.00)	50 (44–60)	53 (45–62)	1006	628	334	44	1590	422	1020	589	372	59	1550	490
Dimitry A (2011)	Non-Asian	Russian	26.9 ± 4.8	28.3 ± 5.9	59.9 ± 7.9	26.9 ± 4.8	767	367	330	70	1064	470	769	333	337	99	1003	535
Jessican (2012)	Non-Asian	American	29.5 ± 7.6	33.7 ± 7.6	48.6 ± 13.0	46.0 ± 12.3	567	105	278	184	488	646	1150	175	547	428	897	1403
Aleksey G Nikitin (2017)	Non-Asian	Russian	28.7 ± 4.8	30.5 ± 5.0	54.4 ± 11.0	60.0 ± 10.2	443	297	124	22	718	168	862	500	293	69	1293	431
Oswald Ndi Nfor (2018)	Asian	Taiwanese women	NA	NA	47.60 ± 10.80	55.56 ± 9.19	8934	3707	4061	1166	11475	6393	974	353	441	180	1147	801
Mariam Al Ali (2019)	Asian	Emirati	NA	NA	NA	NA	264	137	99	28	373	155	153	86	57	10	229	77
Kazem Vatankhah Yazdi (2020)	Asian	Iranian	23.07 ± 1.03	24.00 ± 1.23	65.5 ± 7.3	65 ± 7.5	106	46	50	10	142	70	162	31	104	27	166	158

**Table 2:** Heterogeneity test.

CDKAL1	Group	A fixed-effects model			A random-efforts model			Heterogeneity		
		OR (95% CI)	*Z*	*p*	OR (95% CI)	*z*	*p*	*X* ^2^	*I* ^2^ (%)	PQ test
A vs. C	Total	0.89 [0.86, 0.92]	6.17	*p* < 0.00001	0.85 [0.77, 0.94]	3.25	*p* = 0.001	76.82	83%	*p* < 0.00001
	Asian	0.92 [0.88, 0.97]	3.36	*p* = 0.008	0.87 [0.75, 1.02]	1.73	*p* = 0.08	65.37	89%	*p* < 0.00001
	Non-Asian	0.83 [0.78, 0.88]	5.88	*p* < 0.00001	0.83 [0.77, 0.88]	5.55	*p* < 0.00001	5.59	11%	*p* = 0.35

AA vs. AC+CC	Total	0.77 [0.73, 0.82]	9.15	*p* < 0.00001	0.77 [0.70, 0.85]	5.42	*p* < 0.00001	149.89	92%	*p* < 0.00001
	Asian	0.83 [0.77, 0.88]	7.68	*p* < 0.00001	0.75 [0.64, 0.88]	3.61	*p* = 0.0003	29.52	76%	*p* = 0.0001
	Non-Asian	0.79 [0.72, 0.86]	4.99	*p* < 0.00001	0.79 [0.72, 0.87]	4.97	*p* < 0.00001	84.86	96%	*p* = 0.58

AA+AC vs. CC	Total	0.86 [0.80, 0.92]	4.47	*p* < 0.00001	0.81 [0.69, 0.94]	2.68	*p* = 0.007	33.42	61%	*p* = 0.001
	Asian	0.91 [0.83, 0.99]	2.23	*p* = 0.03	0.85 [0.66, 1.10]	1.21	*p* = 0.23	59.98	86%	*p* < 0.00001
	Non-Asian	0.78 [0.70, 0.87]	4.39	*p* < 0.0001	0.78 [0.70, 0.87]	4.38	*p* < 0.0001	1.70	0	*p* < 0.00001

## Data Availability

The data used to support the findings of this study are included within the article.
